# Dephytinisation with Intrinsic Wheat Phytase and Iron Fortification Significantly Increase Iron Absorption from Fonio (*Digitaria exilis*) Meals in West African Women

**DOI:** 10.1371/journal.pone.0070613

**Published:** 2013-10-04

**Authors:** Yara Koréissi-Dembélé, Nadia Fanou-Fogny, Diego Moretti, Stephan Schuth, Romain A. M. Dossa, Ines Egli, Michael B. Zimmermann, Inge D. Brouwer

**Affiliations:** 1 Department of Food Sciences and Nutrition Group, Division of Human Nutrition, Wageningen University, Wageningen, The Netherlands; 2 Institute of Food, Nutrition and Health, ETH Zurich, Zurich, Switzerland; 3 Institute of Rural Economy (IER), Regional Agronomical Research Center (CRRA) Sotuba, Food Technology Laboratory (LTA), Bamako, Mali; 4 Department of Nutrition and Food Sciences, FSA, University of Abomey Calavi, Cotonou, Benin; 5 Steinmann Institute of Geology, Mineralogy, and Petrology, University of Bonn, Bonn, Germany; Cairo University, Egypt

## Abstract

Low iron and high phytic acid content make fonio based meals a poor source of bioavailable iron. Phytic acid degradation in fonio porridge using whole grain cereals as phytase source and effect on iron bioavailability when added to iron fortified fonio meals were investigated. Grains, nuts and seeds collected in Mali markets were screened for phytic acid and phytase activity. We performed an iron absorption study in Beninese women (n = 16), using non-dephytinised fonio porridge (FFP) and dephytinised fonio porridge (FWFP; 75% fonio-25% wheat), each fortified with ^57^Fe or ^58^Fe labeled FeSO_4_. Iron absorption was quantified by measuring the erythrocyte incorporation of stable iron isotopes. Phytic acid varied from 0.39 (bambara nut) to 4.26 g/100 g DM (pumpkin seed), with oilseeds values higher than grains and nuts. Phytase activity ranged from 0.17±1.61 (fonio) to 2.9±1.3 phytase unit (PU) per g (whole wheat). Phytic acid was almost completely degraded in FWFP after 60 min of incubation (pH≈5.0, 50°C). Phytate∶iron molar ratios decreased from 23.7∶1 in FFP to 2.7∶1 in FWFP. Iron fortification further reduced phytate∶iron molar ratio to 1.9∶1 in FFP and 0.3∶1 in FWFP, respectively. Geometric mean (95% CI) iron absorption significantly increased from 2.6% (0.8–7.8) in FFP to 8.3% (3.8–17.9) in FWFP (*P*<0.0001). Dephytinisation of fonio porridge with intrinsic wheat phytase increased fractional iron absorption 3.2 times, suggesting it could be a possible strategy to decrease PA in cereal-based porridges.

## Introduction

Iron deficiency anemia is a prevalent micronutrient deficiency in Africa. Low iron intake and low bioavailability contribute to its high prevalence [1]. Fonio (*Digitaria exilis*) is an indigenous West African cereal, contributing to household food security, due to the early maturing growth of some varieties [2]. In semi-arid areas, fonio represents a major part of the diet. In sub-humid zones, fonio is stored for long periods, to be used as staple food during a food shortage season [3]. In consumption areas, fonio is mainly consumed as couscous or as porridge made from grain or flour [4]. In urban areas in Mali, fonio has been estimated to be consumed at least once per week by one-third of the women [5].

Iron concentration in processed fonio products is low, being 0.8 to 1.8 mg/100 g on dry weight basis [6]. In addition, as most plant-based foods, fonio grains contain phytic acid (myo-inositol 1, 2, 3, 4, 5, 6 hexakis [dihydrogen phosphate]) which forms complexes with iron, thus reducing its bioavailability [7], [8]. Both iron content and bioavailability in fonio-based meals would need to be increased to contribute meaningfully to the iron requirements. Iron fortification of staple foods has been recommended as a strategy to increase the content of available iron in the foods [9]. Ferrous (Fe^II^) sulfate has been the standard iron compound used in iron absorption studies and the bioavailability of other iron compounds are expressed relatively to its bioavailability [10]. Because iron absorption is strongly inhibited by phytic acid (PA) in cereals-based foods, reducing PA levels appears to be essential for improved iron absorption from iron-fortified plant-based diets [11]. Phytic acid can be degraded by adding microbial phytases or by enhancing the activity of intrinsic phytase present in a large range of plant-derived foods [12], [13]. Addition of microbial phytase immediately before consumption significantly affects the iron bioavailability from an inhibitory test meal as the pure phytase enzyme degrades PA during digestion before it is degraded by peptic enzymes [14]. High apparent phytase activity (PAC) was found in untreated whole grain rye, wheat, triticale, buckwheat and barley [13]. Previous studies have shown that adding wheat to cereal or legume-based complementary food mixtures completely degraded phytate at optimal incubation conditions [15]. However, when using grains as phytase source, an incubation step is likely to be needed as the permanence time of the free phytase in wheat may be too short in the gastro-intestinal tract to meaningfully degrade phytic acid [16], [17]. This study aimed at assessing the effect of PA degradation in fonio through a food-based approach and to quantify its effect on iron bioavailability in humans. We first investigated PA content and PAC of commonly used grains, seeds and nuts. Secondly, we characterised PA degradation in fonio-based porridges using whole grain wheat flour as native phytase source. Thirdly, we measured the effect of phytic acid degradation on iron bioavailability in iron-fortified fonio porridge in human subjects.

## Methods

Three sets of experiments were conducted: 1) analysis of PA content and PAC of grains, seeds and nuts commonly consumed in Mali, 2) PA degradation in fonio porridge using whole wheat flour, and 3) measuring the effect of PA degradation on iron bioavailability from iron-fortified fonio porridge in young women. The experiments were carried out in Mali and Benin, two West African countries. The iron absorption study was registered at http://www.clinicaltrials.gov; ID: NCT01443832

### Grains, Nuts and Seeds

An amount of 500 g of grains, seeds or nuts was purchased in March 2008, each from three different commercial suppliers located at the three main local markets in Bamako (Mali). Fonio paddy grain (*Tamatioi* landrace) was bought from farmers in Tominian village, Ségou, Mali. All grains, seeds or nuts were of whole grain quality without any thermal or chemical treatment, and were dry cleaned to remove impurities, dusts, sands and immature grains.

At the laboratory of food technology (LTA) of the Institute of Rural Economy of Mali (IER), all samples were pooled per grain, nut or seed respectively and homogenously mixed. A sample of 100 g of the pooled Bambara nut, baobab seeds, hibiscus seeds, pumpkin seeds, African locust bean seeds and whole wheat grain was washed with distilled water, dried overnight at room temperature (30°C) under ventilation and packed in a sealed polyethylene plastic bag. Fonio paddy grains were dehulled to have the whole grain fonio, and washed using the standardized traditional method described elsewhere [6]. All samples were stored at −18°C until shipment by courier express to ETH Zurich for analyses. Prior to analysis all grains, nuts or seeds (untreated) were frozen in liquid nitrogen and milled with a centrifugal mill (0.5 mm mesh; Retsch ZM1, Retsch GmbH, Haan, Germany).

The dry matter (DM) content was determined gravimetrically after drying about 1 g milled grains, nuts or seeds for 24 h with forced-air drying oven at 105±3°C.

### Phytase Activity

About 1 g of milled grains, nuts or seeds was added to 20 mL buffer (pH 5.0: 0.2 M acetate buffer; pH 8.0: 0.2 M Tris buffer) containing 7.5 mM PA prepared from PA dodecasodium salt (Sigma- Aldrich Chemie GmbH, Steinheim, Germany). The measurement was performed for 1 h at 45°C under constant stirring with aliquots taken every 20 min. The reaction was terminated by adding 0.5 mL 0.9 M trichloroacetic acid to each 0.5 mL aliquot. The determination of PAC was based on the measurement of liberated inorganic phosphate (IP) from PA in a certain time interval. IP was determined according to the van Veldhoven's and Mannaerts method [18] at 4 time points (0, 20, 40, 60 min). IP was liberated at a constant rate and apparent PAC was calculated by linear regression of the IP determined for each time point. Apparent phytase activity is expressed in phytase units (PU) per g DM of grains, nuts or seeds. One PU is equivalent to the enzymatic activity that liberates 1 µmol inorganic phosphate per min.

### Phytic Acid

Phytic acid was determined according to a modified method of Makower [19] consisting of an extraction and a selective precipitation of phytate in which cerium replaced iron in the precipitation step according to Hurrell et al [16]. The inorganic phosphate liberated from the phytate digestion was measured according to the van Veldhoven's and Mannaerts method [18]. The difference of duplicate sample relative to the mean value was <10%. PA content in mg/100 g DM refers to 100 g freeze-dried porridge sample.

### Phytic Acid degradation in fonio porridge

The conditions for PA degradation were optimized for the grains with the highest PAC which was wheat. Degradation of PA was tested with fonio porridge by adding 25% of whole wheat flour as previously demonstrated in complementary foods [15].

#### Porridge Preparation

A standard fonio porridge recipe was developed at LTA Mali based on the traditional recipe collected during a short food consumption survey performed among a sample of households selected in rural areas in Mali. The standardized traditional recipe was adapted to a previously developed process [15]. For the experiment, 50 g of flour in the proportion of 75% whole fonio flour (37.5 g) and 25% whole wheat flour (12.5 g) was used. To prepare the porridge, fonio flour was mixed into 200 mL of water; the mixture was added to 800 mL of boiling water and cooked for 22 minutes under constant stirring. The porridge was removed from the heat source and the measured pH was around 6. The pH was adjusted to ≈5.0 (optimal condition for PAC) with 1 M citric acid (1.4 mL). No other ingredients were added. The water used for the experiment was purified by reverse osmosis (Nanopure Cartridge System, Skan AG, Basel, Switzerland).

#### Phytic Acid Degradation

The fonio flour porridge prepared as described above was left at room temperature for cooling down to ∼50°C (optimal incubation temperature for PAC). Whole wheat flour was added to the porridge, which was mixed with a blender for about 5 min. The temperature of the mixed porridge reduced to 47–48°C after mixing, so the porridge was heated again to reach an incubation temperature of 50°C. Six aliquots of 10 g were weighed into 100 mL covered polyethylene containers. One aliquot was quickly cooled on ice to restrain wheat intrinsic enzymatic activity, and frozen immediately at −18°C corresponding to time zero. The other five aliquots were put into incubator at 50°C under constant stirring (600 rpm, MEMMERT incubator shaker series, MEMMERT GmbH Co.KG, Schwabach FRG, Germany). Thirty minutes after incubation, and then every 60 min, one aliquot was withdrawn from the incubator, quickly cooled on ice to restrain enzymatic activity, and immediately frozen at −18°C. Temperature and pH were monitored continuously. All samples were kept at −18°C until transport to Wageningen University, Division of Human Nutrition, for PA content analysis as described above.

### Iron Absorption

The iron absorption study was carried out at the Department of Nutrition and Food Sciences, University of Abomey-Calavi in Benin.

#### Subjects

A group of 16 apparently healthy young Beninese women aged 18–30 years (confirmed with birth certificate or other official documents) were recruited between September 2010 and January 2011, from neighborhood communities. Inclusion criteria were: i) body weight <65 kg (measured according to standard procedures [20]), ii) no pregnancy (confirmed by rapid pregnancy test using Nancy HCG kit, 3H Medical Products, China) and no breastfeeding, iii) no reported chronic medical illnesses, iv) no reported symptoms of malaria in the last two months (fever, headache, stomach ache, diarrhoea, nausea, vomiting), v) no recent malaria (negative blood smear response based on Giemsa stained microscopy following standard guidelines [21]), vi) no intake of vitamin and mineral supplements two weeks preceding the study, vii) no blood donation in the last six months, vii) no iron medication or supplementation two weeks before and during the study, ix) no reported allergy to gluten. The subjects represented a population who potentially would use fonio based meals for a certain time period of the year (2–3 months before the harvesting of other cereals), and who are expected to be mildly or moderately iron deficient/anemic.

A sample size of 16 subjects was estimated to be adequate to detect an intra-individual variation in log (iron absorption) of 0.35 [22] and a 30% increase in iron bioavailability [16] with 95% power and a significance level of 5%, accounting for a conservative drop out of 2 participants. Before enrolment, study participants received a full explanation of the study in written form and orally during group discussion sessions. Written informed consent was obtained from all subjects before starting the study. The study protocol was approved by the National Provisional Ethical Committee for Public Health Research in Benin (Comité National Provisoire d'Ethique pour la Recherche en Santé Publique, CNPERS) and by the Medical Ethical Committee of the University of Wageningen (Medisch Ethische Toetsings Commissie, METC-WU).

#### Study Design

**Figure 1:**
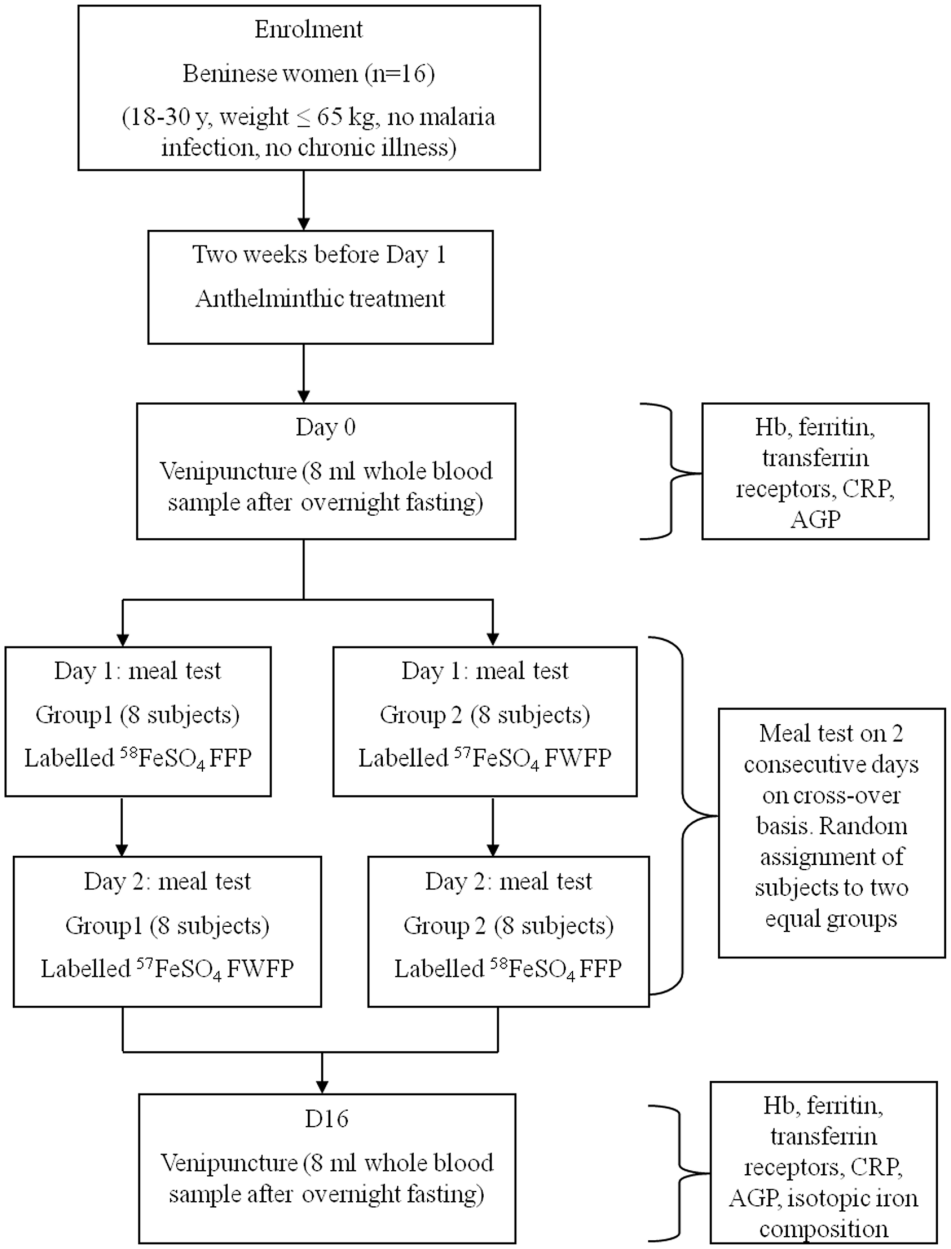
Experimental design of iron absorption test with fonio porridge using stable iron isotope. FFP, Fonio flour porridge; FWFP, fonio + wheat flour porridge; ^57^FeSO_4_ and ^58^FeSO_4_, ferrous sulfate labeled with Fe isotopes ^57^Fe and ^58^Fe, respectively.

Using a crossover design, subjects were given two iron-fortified fonio porridges labeled with ^57^Fe or ^58^Fe, on two consecutive days (Figure 1). Two weeks before the test, participants were dewormed with anthelminth (Zentel, 400 mg Albendazole tablets, laboratoire Glaxosmithkline, France). On day 0, participants were invited to the study location, and weight and height were measured [20].

On day 1 and day 2, the two test meals were randomly received by the participants. On d 0 and fourteen days later, on d 16, a venous blood sample (8 mL) was collected in a K_2_EDTA tubes for Hemoglobin (Hb) concentration measurement, and serum tubes between 7.00 and 8.00 a.m. after overnight fasting (no food after 8.00 p.m. and no drink after 12.00 a.m. on the evening before day 1). During the test period, to ensure that participants were fasted, the evenings before days 1, 2, and 16, participants were lodged in a centre in the neighbourhood of University of Abomey Calavi, and dinner was served from 6.30 to 7.45 p.m.

The test meals were served to participants from 7.00 to 9.00 a.m. and consumed under standardized conditions and close supervision. No food and drink was allowed to the participants within 3 hours after the meal was consumed. After this period each participant received a breakfast package which she was allowed to consume ad libitum.

#### Stable Isotope Labels Preparation and Test Meals

The isotopically enriched ^57^FeSO_4_ and ^58^FeSO_4_ were prepared from isotopically enriched ^57^Fe and ^58^Fe (enrichment 97.5 and 99.6% respectively, Chemigas Boulogne, France) respectively, by dissolution in diluted sulphuric acid (0.1 mol H_2_SO_4_/L). The test meals consisted of two fonio porridges of 240 g: single fonio flour porridge (FFP) and mixed fonio-wheat porridge (FWFP, ratio 3∶1, weight-to-weight). Fonio and wheat flours were made from whole grains. The porridges were prepared in bulk at the Division of Human Nutrition of Wageningen University, following the recipe described above. Each bulk of porridge (FFP and FWFP) was divided into 16 portions of 240 g. FFP portions were immediately cooled on ice. FWFP portions were placed in incubator for 3 h at 50°C under constant stirring at 115 rpm (Innova 44, incubator shaker series, New Brunswick Scientific Co., Inc, Eppendorf Company, USA) after which they were immediately cooled on ice. The porridge portions were stored at −18°C and sent on dry ice by express courier to Benin one week before the study. Each evening before test day 1 and day 2, 16 portions were thawed overnight in a fridge at 4 to 5°C. On the test days, thawed portions were quickly warmed up for 1 minute in a microwave oven (400–1200 W) and 4 mg of iron isotope solutions were added quantitatively about 5 minutes before consumption. Isotope labels were carefully spread on the surface of the test meals. After consuming the entire meal, the participants consumed 50 mL of drinking water used to rinse the bowl.

#### Phytate and Iron Content of the Test Meals

Iron concentration in the test meals was analysed using Inductively Coupled Plasma Atomic Emission Spectrometry (ICP-AES), after microwave digestion with hydrofluoric acid (40%), HNO_3_ (65%) and H_2_O_2_ (30%). Phytate content was determined as described above [18], [19]. Phytate-to-iron molar ratio was estimated as [mg phytate/molecular weight of phytate]/[mg Fe/Fe molecular weight].

#### Blood Analysis and Iron Isotope Measurements

Hemoglobin (Hb) concentration in whole blood was measured immediately after blood collection, using an automated Sysmex counter (KX 21-N, Sysmex, cyanide-free reagent used for Hb detection, normal values range 11.50–15 g/dL). The 3-level Eightcheck-3WP control material provided by the manufacturer was used for quality check. Overall anemia was defined as Hb<12 g/dL, moderate anemia as Hb<10 g/dL and severe anemia as Hb<9.0 g/dL [23]. Serum ferritin (SF), soluble transferrin receptors (sTfR), C-reactive protein (CRP) and α_1_-acid glycoprotein (AGP) were measured simultaneously using an in-house sandwich Enzyme-linked immunosorbent assay (ELISA) technique [24]. All measurements were done in duplicates and measurements showing CVs ≥10% were repeated. The CVs (inter-essay) for the various indicators were: SF, 2.6%; sTfR, 2.4%; CRP, 7.2% and AGP, 2.9%. Certified quality control samples from the CDC/Atlanta and BioRad Liquicheck (BioRad, Munich, Germany) were used. Iron deficiency was defined as SF<12 µg/L or sTfR>8.5 mg/L, and iron deficiency anemia as Hb<12 g/dL and SF<12 µg/L. CRP>10 mg/L indicated ongoing inflammation. AGP values >1 g/L were used to identify existing inflammation.

#### Isotopic Composition in Blood Samples

Whole blood samples were mineralised and separated as described by Schoenberg and von Blanckenburg [25]. Iron isotopic analyses were performed using a high-resolution multicollector-inductively coupled plasma-mass spectrometer (Thermo-Fisher Neptune, University of Bonn, Germany) [26]. Copper was added (1ppm Alfa-Aesar Specpure) to the solution immediately prior to analysis to correct for mass bias [25], [27]. Each isotopically enriched solution was measured in triplicate using the standard-sample bracketing technique [25], [27], [28]. One third of the samples were re-measured as external duplicates. Blank solutions and ^57^Fe or ^58^Fe indicator solutions were used as an external quality control during the measurements.

#### Calculation of Fe Absorption

Calculation of iron absorption was based on the shift in the isotopic ratios after a 14-d incorporation period as described by Walczyk et al [29]. Circulating iron was calculated on the basis of the blood volume, which was estimated from the participant's height and weight [30]. Isotopic ratios were calculated according to the principle of isotope dilution [29] and taking into account that isotopic labels are not monoisotopic [31]. An incorporation rate of 80% of the absorbed iron into red blood cells was assumed for our group of young women [32].

### Statistical Analysis

Data analysis was performed in Excel (Microsoft Office 2007, Microsoft, Seattle, USA), GraphPad Prism version 6.01 for Windows (GraphPad Software, San Diego, CA, USA) and PASW software (version 18.0; IBM SPSS, Chicago). *P* values<0.05 were considered significant. Visual check and Shapiro-Wilk test were used to check for normality of the distribution. Normally distributed data were expressed as mean ± SD, non-normally distributed as median (interquartile range) and as geometric mean ± SD. Fractional iron absorption distribution was log-transformed to normality. Log-transformed fractional iron absorption from FFP and FWFP meals were compared using paired *t* tests. Pearson's correlation was used to test for association between (log) SF and log fractional iron absorption from different meals.

## Results

### Apparent Phytase Activity and Phytic Acid in Grains, Nuts and Seeds

Apparent PAC ranged from 0.2 PU/g DM for fonio to 2.9 PU/g DM for whole wheat (Table 1). The optimal conditions for PAC of grains, nuts and seeds were 50°C and pH≈5. Whole wheat grain (cereal) showed the highest PAC compared to the legume (Bambara nut) and oilseeds (Hibiscus, Baobab, African locust bean and pumpkin seeds), having PAC lower than 1 PU/g DM.

**Table 1:** Apparent phytase activity and phytic acid content of untreated grains, nuts and seeds.

Grains, nuts and seeds common name	Botanical name	Phytase activity^1^ (PU^2^/g dry matter)^3^	Phytic acid (g/100 g dry matter)^4^
**Cereals**			
Fonio	*Digitaria exilis*	0.17±1.61	0.89
Wheat	*Triticum aestivum*	2.88±1.27	0.85
**Legumes**			
Bambara nut	*Vigna subterranea*	0.65±7.25	0.39
**Oilseeds**			
African locust bean	*Parkia biglobosa*	0.54±0.95	0.46
Baobab	*Adansonia digitata*	0.28±3.46	1.80
Hibiscus	*Hibiscus sabdariffa*	0.86±7.13	1.16
Pumpkin	*Cucurbita pepo*	0.21±5.08	4.26

1 Measured at pH 5.0, 45°C.

2 1 phytase unit (PU) is equivalent to the enzymatic activity that liberates 1 µmol inorganic phosphate per min under specified conditions.

3 Values represent mean ± SD of triplicate analysis in dry products.

4 Values are mean of duplicate analysis in wet products.

Phytic acid in all the untreated grains, nuts and seeds ranged from 0.4 g/100 g DM in Bambara nut to 4.3 g/100 g DM in pumpkin seed, with higher levels in oilseeds (except African locust bean) compared to cereals and legumes (Table 1). The PA content in cereal grains was in the same range while apparent differences were observed among the oilseeds.

### Phytic Acid Degradation in Fonio Porridge

**Figure 2:**
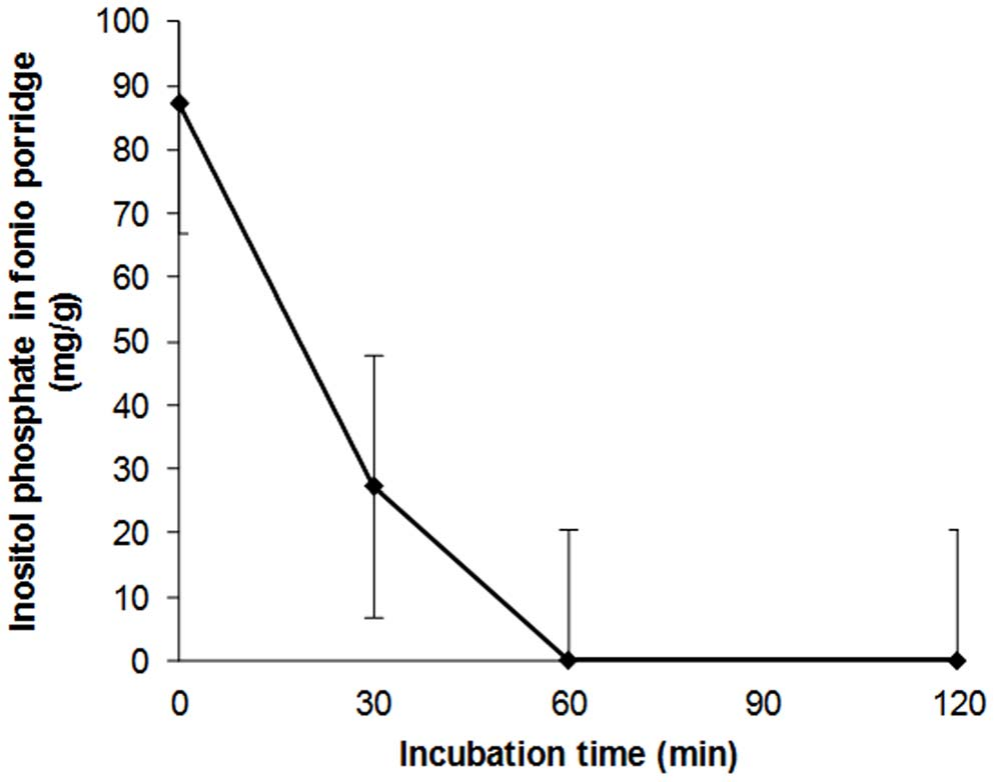
Phytic acid (IP6) content in fonio (75%) - wheat (25%) flour porridge at different incubation time (0 to 240 min).

Figure 2 shows PA degradation in porridge with the addition of 25% of whole grain wheat. Phytic acid decreased from 87.5 g/100 g DM at 0 min of incubation to 27.5 g/100 g DM at 30 min of incubation, corresponding to 30% of reduction. After 1, 2 and 4 hours of incubation, PA concentration was 0.18 g, 0.12 g and 0.11 g, respectively, indicating that removal of PA was almost complete after 1 hour of incubation (50°C, pH≈5).

### Participants in Iron Absorption Study

Sixteen participants were recruited, but one did not complete the test because suffering from malaria on day 16. Participants were aged 24 years-old on average, with a mean (±SD) body mass index (BMI) of 21.2±3.3. Mean (±SD) hemoglobin and body iron stores concentration was 11.9±1.4 g/L and 3.5±4.9 mg/kg, respectively. Median (25^th^–75^th^) ferritin and transferrin receptor was 22.9 (12.7–74.8) µg/L and 6.0 (5.2–7.6) mg/L, respectively. Seven subjects had moderate anaemia. Three subjects had iron deficiency anemia and one subject was iron deficient but not anemic. Mean (±SD) CRP and AGP concentration was 3.4±7.8 mg/L and 0.8±0.2 mg/L, respectively. The CRP and AGP concentration was elevated for two (14.7 and 28.2 mg/L) and three participants (1.05, 1.04 and 1.06 g/L) respectively.

#### Test Meals

Iron concentration in the portion (240 g) of fonio flour porridge and fonio-wheat flour porridge was respectively 0.34 mg and 0.51 mg. Mean (±SD) phytate concentration in FFP and FWFP was respectively 96.0±16.2 mg and 16.2±4.4 mg. The phytate∶iron molar ratio decreased from 23.7: 1 in FFP to 2.7∶1 in FWFP. The non dephytinised fonio porridge meal with iron fortification showed a phytate∶iron ratio of 1.9∶1, which decreased to 0.3∶1 in FWFP meal (Table 2).

**Table 2:** phytate, phytate∶iron molar ratio and iron absorption from high phytate (without wheat) and low phytate (with wheat) iron-fortified fonio porridges.

Fonio porridges	Phytate content in portion (mg, wet weight basis)	Phytate-to-iron molar ratio of fonio prorridges	Phytate-to-iron molar ratio of iron fortified fonio prorridges	Iron absorption (%)^1^
**Without wheat**	96.0±16.2	23.7∶1	1.9∶1	2.6 (0.8, 7.8)^2^
**With wheat**	16.2±4.4	2.7∶1	0.3∶1	8.3 (3.8, 17.9)^2^

1 Geometric mean (−SD, +SD).

2 Values are significantly different (*P*<0.0001).

#### Iron Absorption

**Figure 3:**
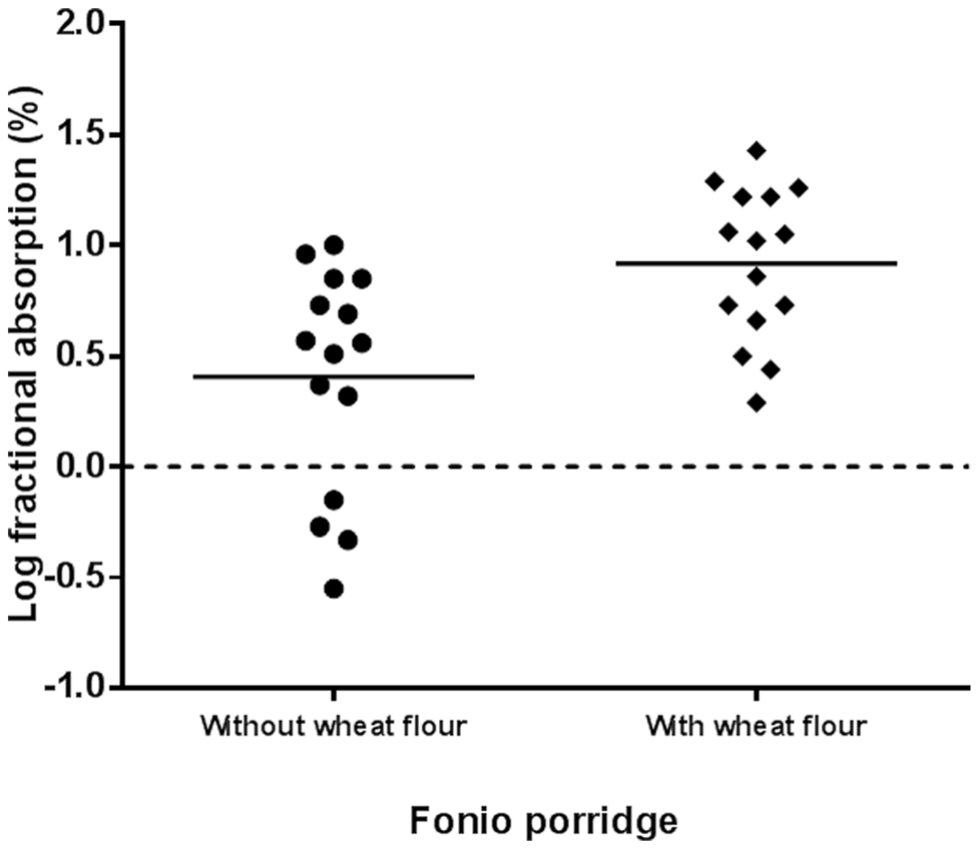
Comparison of (log) fractional iron absorption between non-dephytinised (without wheat flour, FFP) and dephytinised (with wheat flour, FWFP) fonio porridge (n = 15). Black lines show mean log fractional iron absorption from each meal. Geometric mean (95% CI) of iron absorption ratio from FFP and FWFP is 2.6 (0.8–7.8) and 8.3 (3.8–17.9) respectively. Log fractional iron absorption between meals significantly different (paired *t* test, *P*<0.0001).

Geometric mean (95% CI) iron absorption from FFP and FWFP meal was 2.6% (0.8–7.8) and 8.3% (3.8–17.9) respectively (Figure 3). Fractional iron absorption from FWFP was 3.2 times higher compared to FFP (paired *t* test, *P* < 0.0001).

## Discussion

### Phytic Acid Content and Apparent Phytase Activity of Grains, Nuts and Seeds

Phytic acid was found at different concentrations in all analyzed grains, nuts and seeds. Previous studies also showed PA in grains and legumes to vary depending on the type of plant-based food, growing conditions, harvesting techniques, processing methods, analytical methods and the harvesting year [33], [34]. The PA concentration of 0.85 and 0.89 g/100 g DM reported on cereals (wheat and fonio) in our study is slightly lower compared to 0.98 on whole wheat [35] and 1.03 reported in untreated wheat grain and in the range of 0.79 to 1.63 g/100 g DM reported for cereals grains [13]. Our results confirm previous studies showing high PA in oilseeds (1.00 to 2.22 g/100 g DM) compared to legumes (0.48 to 1.40 g/100 g DM) [13], [35], although we report a high value of 4.26 g/100 g DM in pumpkin seeds. The 0.46 g/100 g DM of African locust bean and 1.80 g/100 g DM of baobab seed reported in our study were higher than the 163 and 6.6 mg/100 g DM respectively reported earlier [36], [37]. These differences could be due to differences in the analytical methods used for PA determination as there is no international agreed upon standardized method for its determination.

Our PA analytical method is best suited to quantify IP6 and IP5. Other inositol phosphates like IP4, IP3 and IP2 were not detected, however they are considered to have less negative influence on iron bioavailability as they do not form strong complexes with minerals and trace elements [38], [39].

Apparent PAC was very low in our untreated seeds and nuts compared to grains, especially wheat. These results confirm previous studies showing high PAC (1.8 to 6.9 PU/g DM) in some cereals such as rye, triticale, wheat and barley and PAC comparable to fonio in others such as maize, millet, oat, rice, sweet maize, and sorghum (0.1 to 0.4 PU/g DM) (13, 40). The 2.9 PU/g DM of wheat reported in our study is similar to 3.1 PU/g DM reported earlier [13]. The PAC in our grains, seeds and nuts was relatively low (except for wheat grain) maybe because our samples were not soaked, germinated or fermented before being analysed. These traditional food processing methods are known to activate phytase in grains, legumes and seeds, and therefore decrease PA content in plant foods [40], [41]. The high PAC of whole wheat grain could provide sufficient enzymatic activity to degrade PA in mixtures of cereals or legumes [13]. Phytases from different sources are likely to differ in sensitivity to substrate inhibition and temperature and pH optima [13]. In most studies, phytases are isolated from grains, nuts and seeds and sometimes purified before determining the enzymatic activity [42], [43]. In our study, no relationship was observed between PAC and PA content of the untreated grains, nuts and seeds, indicating that the high PAC of cereals, such as wheat, is not associated with high PA content. This was already reported in a study on rye and wheat [13]. Our results confirm that whole wheat flour could be used as a source of natural phytase to produce low PA containing fonio porridge, and suggest its use in other more micronutrient rich legume and cereal mixtures.

### Phytic Acid Degradation

The degradation of PA in fonio porridge by using 25% whole grain wheat flour as a natural phytase source was almost complete after 1 hour of incubation at 50°C with pH of 5.0. This result is in agreement with a previous study showing that adding whole wheat grain to cereal-based complementary foods almost completely degraded PA in a relatively short time [15]. Deviations from the temperature and pH resulted in much less effective PA degradation (data not shown). Most phytases have an optimal pH in the range of 4.5–6.0 and a temperature range of 45–60°C. Outside the optimal range of pH and temperatures the action of phytase is reduced [44]. Application of the method for PA degradation in cereal/legume based weaning foods using phytase naturally occurring in whole wheat grain flour would include the adaptation to household level or to small scale industries producing cost effective weaning foods, especially in developing countries where commercial weaning foods are not affordable or not available, and infants are often fed porridges based specifically on cereals or mixed with legumes containing high amounts of PA. Wheat is readily available worldwide and could be added to porridges as phytase source. After cooking the porridge for example, 25% of whole grain wheat flour could be added, the pH adjusted to ≈5 by adding acidic fruits juices (as usually done at household level) and kept warm during two hours. The porridge would likely require an additional heating step before consumption to infants, or the maintenance of strict hygiene practices. At household level, the addition of wheat would not substantially change the taste of the porridges, indicating that acceptability would not be a major problem. However, the optimal conditions for complete phytic acid degradation in cereal/legume mixtures and the microbiological quality of the products would need to be further investigated in field studies.

### Effect of Phytic Acid Degradation on Iron Bioavailability in Iron-Fortified Fonio Porridge

Dephytinisation with intrinsic wheat phytase reduced phytate-to-iron molar ratio from 24∶1 to 3∶1, while iron fortification decreased the molar ratio to 0.3∶1, and dephytinisation plus fortification increased iron absorption from 2.6% to 8.3% in fonio porridge.

Inhibition of iron absorption by phytate in plant-based meals is dose-dependent at very low concentrations of 2–10 mg per meal [9]. Preferably, the phytate∶iron molar ratio should be lower than 0.4∶1 to achieve meaningful iron absorption when no enhancers of iron absorption are added to the meal [45]. The ratios of 23.7∶1 reported in this study was higher than the range of 12–21∶1 previously estimated for sorghum flour porridges consumed in Benin [46], and much higher than the 5∶1 and 4∶1 reported respectively for wheat flour porridge and rice flour porridges in developing countries [47]. These high values above 0.4 highlight the importance of the inhibitory effect of phytate on iron absorption. This is emphasized in our study by the fact that 4 mg Fe sulfate of fortification of fonio meals without dephytinisation resulted in phytate-to-iron ratio of 1.9∶1, and a mean iron absorption ratio of 2.6%. This would provide only 7.6% of the 1.46 mg/day median absolute requirement for iron absorbed among menstruating non-pregnant non-lactating women [48]. This also confirms that fortification alone is not sufficient to overcome the negative effect of phytic acid in cereal-based foods [11].

Fortification combined with an almost complete reduction of PA by wheat phytase induced a 3.2 fold iron absorption increase from fortified fonio in humans. It has been previouly shown that dephytinization causes a 2 fold increase in absorption from low-tannin Sudan sorghum porridges, but phytate degradation was achieved with commercial phytase [49]. Also, significant increases in fractional absorption of iron (1.7 fold) and zinc (1.5 fold) in adults have been previously reported for both dephytinized cereal-based foods using purified phytase and wheat intrinsic phytase respectively, but without fortification [14], [50]. Previous studies on the inhibitory effect of phytate have also reported a 2–12 fold increase of iron absorption from different dephytinised meals [16], [51], [52]. Together with our results, this adresses the double issue of the low level of bioavailable iron in cereal-based meals caused by the negative effect of phytate on absorption, and the low content of native iron particularly in fonio meals due to losses during processing.

A high level of fortification (4 mg ferrous sulfate/0.5 mg native iron in 24 g dry weight fonio flour) has been used to modify phytate-to-iron ratio in fonio porridges. A practical issue in the context of low income countries is that such a fortification level can be very costly and difficult to apply. Particularly in mass fortification, this level may be too high in view of the amount of fonio consumed. However, this high level may be relatively close to other applications such as home fortification of specific foods which has the advantage to be cost-effective, while adressing the needs of a clearly defined group of the population [10], [52]. In addition, products are usually fortified in their final form, immediately before consumption, limiting the risk of changes in sensory properties [10]. Such fortification form may be suitable for increasing iron content in fonio meal, and should be further explored for assessing cost and technological feasability. While implementing the incubation of fonio porridge and wheat may be feasible in principle at the household level, we did not investigate the effect of direct addition of wheat flour to a fonio meal at the time of consumption as was done by Troesch et al [14] with purified free phytase. Intrinsic wheat phytase would need to be released from its food matrix during digestion and would not interact with food PA if not for a short time period during digestion, likely having little effect on phytic acid content. Germinating and pre-digesting the wheat grains may reduce the need for incubation and may provide a source of readily available natural phytase, which would potentially be active in the gastro-intestinal tract. Such approaches need to be further investigated.

The stable isotope study was based on a single meal design, which could have overemphasized the effect of PA reduction on iron absorption [9]. Cook et al [53] previously reported significantly lower increase in iron absorption from total diet (2.5 fold) compared to increase from a single meal (5.9 fold). In addition, we cannot exclude that other unknown components of wheat flour might have caused the increased iron absorption. However, PA is the main inhibitor of iron absorption [9] and the significantly reduced level of phytic acid means that the natural phytase in wheat is most likely responsible for the improvement in iron absorption.

## Conclusion

Reducing PA content in cereal-based foods is challenging in low-income countries because typical home processing practices often do not achieve a sufficient PA degradation to significantly improve iron absorption. In addition, exogenous phytases are often costly and their availability limited in rural communities. Therefore, the use of locally available and widely used cereals as wheat for dephytinisation needs to be further explored in developing countries with other traditional cereal-based porridges and legume-cereal mixtures. Our results also demonstrate that iron bioavailability from fortified fonio meals was significantly improved by an almost complete reduction of phytic acid by wheat phytase. Dephytinisation using intrinsic wheat phytase could be a promising processing practice to improve iron bioavailability and fortification is required to increase the amount of absorbed iron from fonio meals. The feasability of this processing technique with regard to users' compliance needs to be further explored in developing countries household conditions.
